# The Synaptonemal Complex Protein Zip1 Promotes Bi-Orientation of Centromeres at Meiosis I

**DOI:** 10.1371/journal.pgen.1000771

**Published:** 2009-12-11

**Authors:** Mara N. Gladstone, David Obeso, Hoa Chuong, Dean S. Dawson

**Affiliations:** 1 Cell Cycle and Cancer Biology, Oklahoma Medical Research Foundation, Oklahoma City, Oklahoma, United States of America; 2 Program in Molecular Microbiology, Sackler School of Biomedical Science, Tufts University, Boston, Massachussetts, United States of America; 3 Department of Cell Biology, University of Oklahoma Health Sciences Center, Oklahoma City, Oklahoma, United States of America; Stowers Institute for Medical Research, United States of America

## Abstract

In meiosis I, homologous chromosomes become paired and then separate from one another to opposite poles of the spindle. In humans, errors in this process are a leading cause of birth defects, mental retardation, and infertility. In most organisms, crossing-over, or exchange, between the homologous partners provides a link that promotes their proper, bipolar, attachment to the spindle. Attachment of both partners to the same pole can sometimes be corrected during a delay that is triggered by the spindle checkpoint. Studies of non-exchange chromosomes have shown that centromere pairing serves as an alternative to exchange by orienting the centromeres for proper microtubule attachment. Here, we demonstrate a new role for the synaptonemal complex protein Zip1. Zip1 localizes to the centromeres of non-exchange chromosomes in pachytene and mediates centromere pairing and segregation of the partners at meiosis I. Exchange chromosomes were also found to experience Zip1-dependent pairing at their centromeres. Zip1 was found to persist at centromeres, after synaptonemal complex disassembly, remaining there until microtubule attachment. Disruption of this centromere pairing, in spindle checkpoint mutants, randomized the segregation of exchange chromosomes. These results demonstrate that Zip1-mediated pairing of exchange chromosome centromeres promotes an initial, bipolar attachment of microtubules. This activity of Zip1 lessens the load on the spindle checkpoint, greatly reducing the chance that the cell will exit the checkpoint delay with an improperly oriented chromosome pair. Thus exchange, the spindle checkpoint, and centromere pairing are complementary mechanisms that ensure the proper segregation of homologous partners at meiosis I.

## Author Summary

Meiosis is a specialized cell division that halves the chromosome number and results in the production of gametes. In humans, meiosis normally produces gametes containing exactly one copy of each chromosome. Meiotic errors lead to gametes with incorrect chromosome numbers, a major cause of birth defects and infertility. A key step in meiosis (meiosis I) is the separation of homologous chromosomes. Homologous chromosomes first become physically linked by recombination, which keeps them together until they attach properly at their centromeres to the apparatus that will pull them to opposite sides of the cell. In this study we have used budding yeast to identify processes, beyond recombination, that contribute to meiotic fidelity. We have found that a protein, Zip1, mediates the pairing of chromosome centromeres in a way that greatly enhances the chance they will be properly separated in meiosis, thus preventing the formation of gametes with incorrect chromosome numbers.

## Introduction

The proper segregation of homologous chromosomes at meiosis I depends upon the ability of the partners to attach to microtubules that radiate from opposite poles of the spindle. These microtubules will mediate the separation of the partners at anaphase I. Crossovers promote bipolar attachment of homologous chromosomes to the spindle by creating a link between the partners, allowing them to attach to the spindle as a unit. Recombination is accompanied by assembly of the synaptonemal complex (SC), a structure that tightly aligns chromosomes from end-to-end. Later in meiosis (diplotene) the SC is lost and the homolog pair (termed a bivalent) remains tethered by chiasmata, the connections formed by the crossovers (reviewed in [1]). Proper attachment of the homologous kinetochores to opposite poles of the meiotic spindle creates tension across the bivalent; this tension serves to stabilize kinetochore-microtubule interactions (reviewed in [2]). Bivalents in which only one kinetochore has attached to microtubules, or in which both kinetochores have attached to the same spindle pole, can undergo cycles of microtubule release and re-attachment until a proper spindle orientation has been achieved. During this process, the spindle checkpoint promotes a meiotic delay that blocks anaphase until all the chromosomes are properly attached. However, the meiosis I delays that are triggered by the spindle checkpoint do not always provide sufficient time to allow proper spindle attachment of errant chromosomes. In both mice and yeast, meiotic cells sometimes proceed to anaphase even if one chromosome pair has failed to become properly oriented [3]–[5]. Thus, mechanisms that act to promote a correct initial attachment of microtubules to homologous kinetochores, that will not require re-orientation, should reduce the demand for spindle checkpoint mediated delays and promote meiotic segregation fidelity.

At the time of microtubule attachment, homologous partners are typically linked by chiasmata, which can often be a considerable distance from the kinetochores. If these chiasmata were the only connections between the homologs then the kinetochores of the bivalent might be expected to have rotational freedom such that they could at times face the same spindle pole, which could result in monopolar spindle attachments [6],[7]. However, early observations of the microtubule attachment process demonstrated that the initial attachments of microtubules to kinetochores are usually correct (bipolar). This led Östergren to suggest that the homologous kinetochores must not behave independently, but be arranged, or interact in some way, that orients them toward opposite spindle poles [8].

One opportunity for communication between the homologous kinetochores is during synapsis. Here the homologous centromeres are juxtaposed, but whether they are actively paired has been hard to establish. The most compelling evidence for active pairing of homologous centromeres has come from studies of non-exchange chromosomes (reviewed in [7]). In *Drosophila* females, fission yeast and budding yeast, the centromeres of non-exchange chromosomes pair late in meiotic prophase in a manner that promotes proper disjunction at meiosis I, even in the absence of any obvious interactions along the chromosome arms [9]–[12]. In budding yeast, centromeres also undergo a period of centromere pairing early in meiotic prophase before homologs become aligned with their partners. This pairing is dependent upon the protein Zip1 [13], which is a major component of the synaptonemal complex that zippers homologs together after the initiation of homologous recombination [14]. Like the later stage of centromere pairing in pachytene, which promotes the disjunction of non-exchange partners, this early stage of centromere pairing is homology independent; the pairing process appears to be driven by interactions between proteins at the centromere regions rather than DNA homologies. How the early and late periods of centromere pairing relate functionally is unclear. Also unknown is whether the pairing that can be observed between the centromeres of non-exchange chromosomes reflects a process that also occurs between the centromeres of exchange chromosomes. The fact that non-exchange chromosome pairs are rare in budding yeast [15] has suggested that the centromere pairing observed to drive their segregation might be a process that is used in every meiosis to orient the centromeres of exchange partners [6],[7]. Here we show that pairing of centromeres of non-exchange chromosomes, in late meiotic prophase, requires Zip1, to promote their bipolar attachment to the meiosis I spindle. Moreover, Zip1 was also found to pair the centromeres of exchange chromosomes in pachytene and to persist at centromeres until they begin attaching to microtubules. The results support the model that centromere pairing acts early in the microtubule attachment process to promote an initial, bipolar, attachment of homologous centromeres to the meiosis I spindle, while the spindle checkpoint and exchanges act later to mediate reorientation of any improperly attached partners. Meiotic centromere pairing, exchanges, and the spindle checkpoint appear to act as independent mechanisms that together promote segregation fidelity in meiosis I.

## Results

### Zip1, Zip2, and Zip3 are required for pairing centromeres of non-exchange chromosomes in pachytene

**Figure 1:**
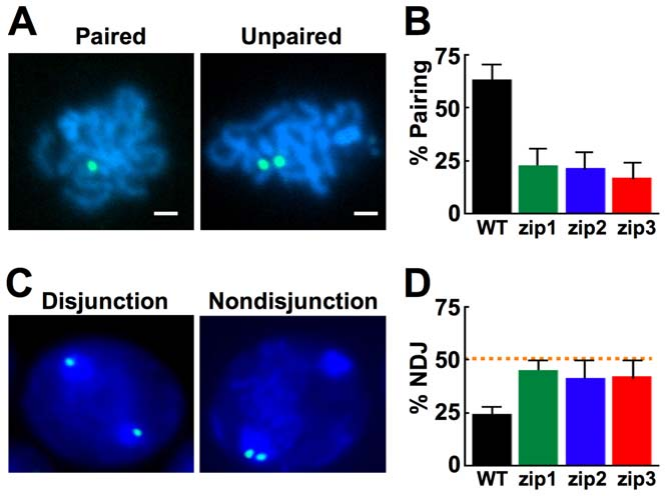
Zip1p, Zip2p, and Zip3p are required for non-exchange chromosome centromere pairing and segregation. (A,B) Isogenic strains carrying a GFP-tagged non-exchange chromosome *V* pair was induced to enter meiosis at 23°C. Thirteen hours following meiotic induction, chromosome spreads were prepared. Spreads with worm-like condensed chromosomes were scored by indirect immunofluorescence for pairing of the GFP dots. Dots that were less than 0.6 µm, center-to-center, were scored as “paired.” (A) Examples of paired and unpaired GFP dots. DAPI staining is shown in blue, GFP in green. Size bar is 1 µm. (B) Quantification of centromere pairing in WT (DMS372; n = 123), *zip1* (DMS382; n = 115), *zip2* (DD732; n = 112), and *zip3* strains (DD737; n = 101). (C,D) Segregation of non-exchange chromosome *V*'s. Cells harvested from meiotic cultures (23°C, T = 17 post meiotic induction) were quickly fixed in ethanol, stained with DAPI and scored for the segregation of GFP-tagged non-exchange chromosome *V*'s in binucleate cells. (C) Examples of binucleate cells in which chromosome *V*'s have disjoined or non-disjoined. (D) Quantification of the percentage of non-disjunction for WT (DMS372), *zip1* (DMS382), *zip2* (DD732), and *zip*3 (DD737) strains.

Centromere pairing occurs at two stages of yeast meiosis. In both stages, centromere pairing is homology-independent. In an early stage of meiotic prophase, before the synaptonemal complex (SC) has been formed [13], centromeres engage in a period of pairing with apparently random partners. Later, at pachytene with full SC [12] when the centromeres of homologous chromosomes lie next to each other, the centromeres of non-exchange chromosome partners pair in a homology-independent fashion. The early stage of centromere pairing requires the SC protein, Zip1 [13]. Here, we used a centromere pairing assay to determine whether the pairing of non-exchange chromosome centromeres in pachytene is also dependent on Zip1 (Figure 1A). In order to obtain cells with a non-exchange chromosome pair, one copy of chromosome *V* was replaced with a homeologous chromosome *V* from *S. carlsbergensis*. These homeologous partners almost never experience crossovers yet disjoin in about 75–90% of meioses [16]; their disjunction driven by homology-independent pairing at their centromeres in meiotic prophase [12]. Each of the non-exchange chromosome *V* partners had an array of lac operator sequences inserted near its centromere, and the cells expressed a lacI-GFP hybrid protein that could localize to the array, providing a GFP-tag that marks the position of that centromere in fluorescence microscopy experiments [12]. Chromosome spreads prepared from wild-type and *zip1* mutant strains were screened, using DAPI staining of the DNA, to identify those with the highly condensed chromosomes typical of late meiotic prophase. These spreads were then scored for the pairing of the GFP-tagged non-exchange chromosome centromeres. Wild-type cells exhibited about 60% pairing of the non-exchange chromosome centromeres (Figure 1B), consistent with earlier studies [12]. In *zip1* mutants the level of pairing was significantly reduced, to the baseline level of about 20% typical of this assay (Figure 1B) [12]. This baseline value of 20% cells with one GFP focus is also the level of pairing observed between two heterologous GFP-tagged chromosomes, each with its own homologous partner [12]. This background level of apparent centromere pairing (one GFP dot) might result from the persistence of centromere clustering from meiotic entry [17],[18] or low levels of pairing promoted by the weak dimerizing properties of GFP-lacI protein [19]. Chromosome spreads with a single GFP focus might also reflect loss of, or failure to detect, the second *lac* operator array. Control experiments (Materials and Methods) were performed to test this. In both wild-type and *zip1* strains the GFP-tagged chromosomes were found to be undetectable in about 5% of chromosome spreads. Thus about 10% of the chromosome spreads scored as “paired” probably had an undetectable GFP tag. This suggests that the *zip1* mutant chromosome spreads have a baseline of about 10% pairing. Whether this reflects biologically meaningful Zip1-independent pairing of the centromeres or an artifact of the approach is not clear.

To test whether the pairing of non-exchange chromosome centromeres in late meiotic prophase requires synaptonemal complex assembly factors beyond Zip1, we assayed the pairing of non-exchange chromosomes in strains deleted for the *zip2* and *zip3* genes. Zip2 and Zip3 are proteins required for assembly of the synaptonemal complex [20]–[22]. In both cases pairing was reduced to the same low levels observed in *zip1* mutants (Figure 1B).

### Zip1, Zip2, and Zip3 are required for non-exchange chromosome disjunction in meiosis I

The pairing of the centromeres of non-exchange partners in pachytene has been correlated with their subsequent disjunction at anaphase I [12]. If centromere pairing of the non-exchange partners promotes their disjunction, then mutations that disrupt the centromere pairing should reduce the levels of disjunction as well. To test this, we assayed the segregation of the GFP-tagged non-exchange chromosomes in cells that had completed meiosis I. The wild-type strains exhibited about 25% non-disjunction in these experiments (Figure 1D). This is slightly higher than the non-disjunction frequency observed for this non-exchange chromosome pair in our previous experiments [16] and is likely due to the reduced incubation temperature (S. Cartinhour, unpublished observations). Deletion of *zip1* reduced the segregation fidelity, resulting in 45% non-disjunction (Figure 1D). Like *zip1* mutants, *zip2* and *zip3* mutants exhibit nearly random segregation of the non-exchange partners in meiosis I (Figure 1D). Thus the absence of late prophase centromere pairing in *zip1*, *2* and *3* mutants is correlated with a nearly complete loss of segregation fidelity of the non-exchange partners. The fact that all three mutants show slightly less than random segregation could reflect a limitation in scoring properly every non-disjunction, or could hint that there are factors beyond the Zip1 proteins that can promote the disjunction of non-exchange partners.

### Zip1 localizes to paired and unpaired non-exchange chromosome centromeres at pachytene

**Figure 2:**
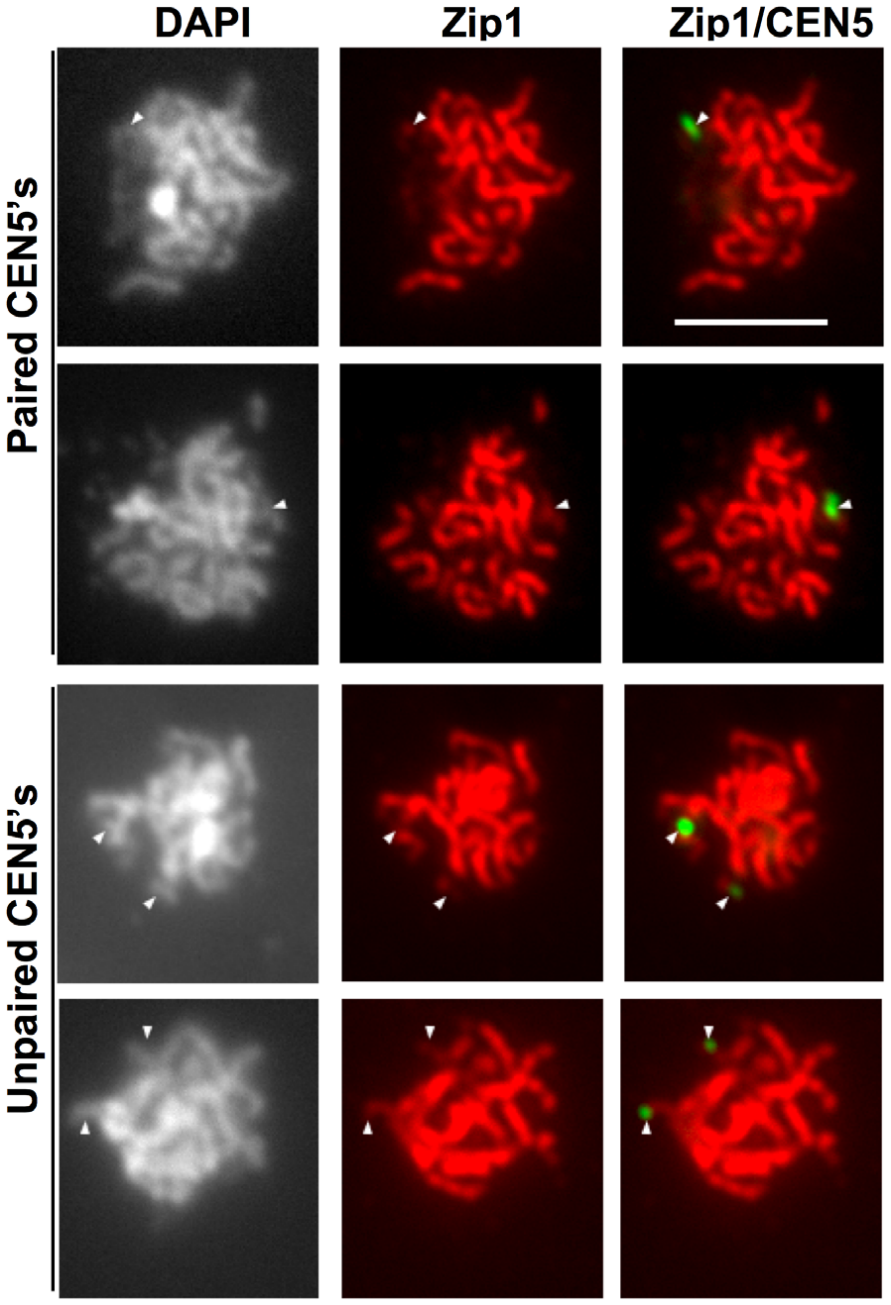
Zip1p localizes to both paired and unpaired non-exchange chromosomes. A strain (DD728) carrying a GFP-tagged non-exchange chromosome *V* pair was induced to enter meiosis at 23°C. Thirteen hours following meiotic induction, chromosome spreads were prepared. Zip1p morphology and the pairing of GFP-tagged centromeres of non-exchange chromosomes were evaluated using indirect immunofluorescence microscopy. Shown are representative chromosome spreads with continuous Zip1 staining. Both paired and unpaired non-exchange chromosomes were typically decorated with Zip1p. Size bar equals 5 µm.

Cells that are engaged in the global, homology-independent, pairing of centromeres in early meiosis exhibit punctate Zip1 foci, some of which localize to paired centromeres – suggesting that the required function for Zip1 in this process is at the centromeres [13]. This raises the question of whether Zip1 is at the centromeres in late prophase to mediate the pairing of centromeres of non-exchange chromosomes. Evaluating the co-localization of Zip1 to specific loci in pachytene cells is complicated by the abundance of Zip1 on the chromosomes and the failure of a particular chromosome to separate from the others in a given spread. Nonetheless, in some chromosome spreads the non-exchange chromosomes are somewhat separated from the bulk of the chromosomes and it is possible to evaluate whether the non-exchange chromosome centromere regions (marked by GFP tags) co-localize with Zip1. For both paired and unpaired non-exchange chromosomes, the centromeres normally were associated with a line of Zip1 staining. However, the Zip1 staining in the area of the GFP-tagged non-exchange chromosome centromeres was not usually as intense as the bright, well-defined lines of Zip1 marking synapsed homologs (Figure 2).

**Figure 3:**
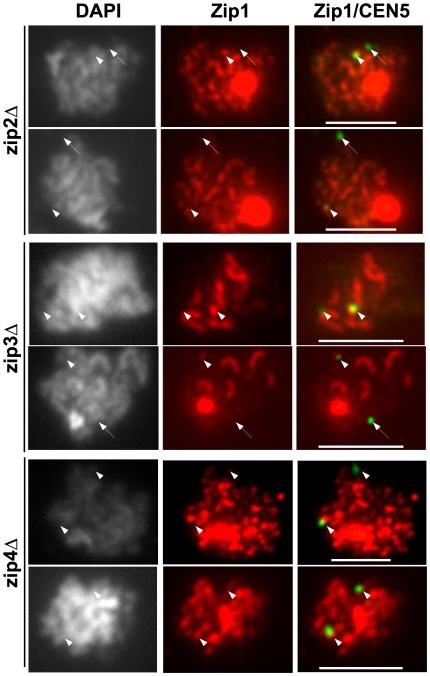
Zip1p association with non-exchange chromosome centromeres in *zip2*, *zip3*, and *zip4* mutants. Isogenic strains carrying a GFP-tagged non-exchange chromosome *V* pair were induced to enter meiosis at 23°C (*WT*, DD728; *zip2*, DD732; *zip3*, DD737; *zip4*, DHC49). Thirteen hours following meiotic induction, chromosome spreads were prepared. Zip1 morphology and the pairing of GFP-tagged centromeres of non-exchange chromosomes were evaluated using indirect immunofluorescence microscopy. The top two rows show *zip2* spreads, the middle two rows show *zip3* spreads, and the bottom two rows show *zip4* spreads. Arrowheads indicate positions of the GFP-tagged centromeres that co-localize with a bright Zip1 signal, arrows indicate GFP-tagged centromeres that do not co-localize with a bright Zip1 signal. Size bars indicate 5 µm.

The requirement for Zip1 for non-exchange disjunction, its localization to centromeres, and the low fidelity of non-exchange segregation in *zip2* and *zip3* mutants raises the question of whether Zip1 is localized to the centromeres of the non-exchange chromosomes in mutants with disrupted SC assembly. We tested this in *zip2*, *zip3* and *zip4* mutants. In all three mutants, SC assembly is defective [23]–[25]. The association of Zip1 with chromosomes does not require Zip2, Zip3, and Zip4. Rather, these proteins are necessary for the ordered assembly of Zip1 into the synaptonemal complex [23]–[25]. Zip1 staining in *zip2*, *zip3*, and *zip4* strains was like that reported previously [23]–[25] (Figure 3). *zip3* exhibited limited stretches of continuous Zip1 staining and a weaker global association of Zip1 on the chromosomes. Zip2 and Zip4 act together to promote synapsis [25] and *zip2* and *zip4* mutants show similar Zip1 staining patterns, with abundant Zip1 foci and little development of continuous SC (Figure 3). In chromosome spreads from *zip3* mutants, the centromeric GFP foci of the non-exchange chromosome were not always associated with a bright focus of Zip1 staining (Figure 3, arrows). In chromosome spreads from *zip2* and *zip4* mutants, co-localization of Zip1 and the centromeres was more difficult to ascertain. With the abundance of Zip1 foci in these spreads, the centromeres were usually adjacent to, or co-localized with, Zip1 foci (Figure 3, arrowheads) though, here too, examples of centromeres that were apparently separated from a Zip1 focus could be seen (Figure 3, arrows).

### Zip1 association with centromeres in pachytene is independent of Zip2 and Zip3

**Figure 4:**
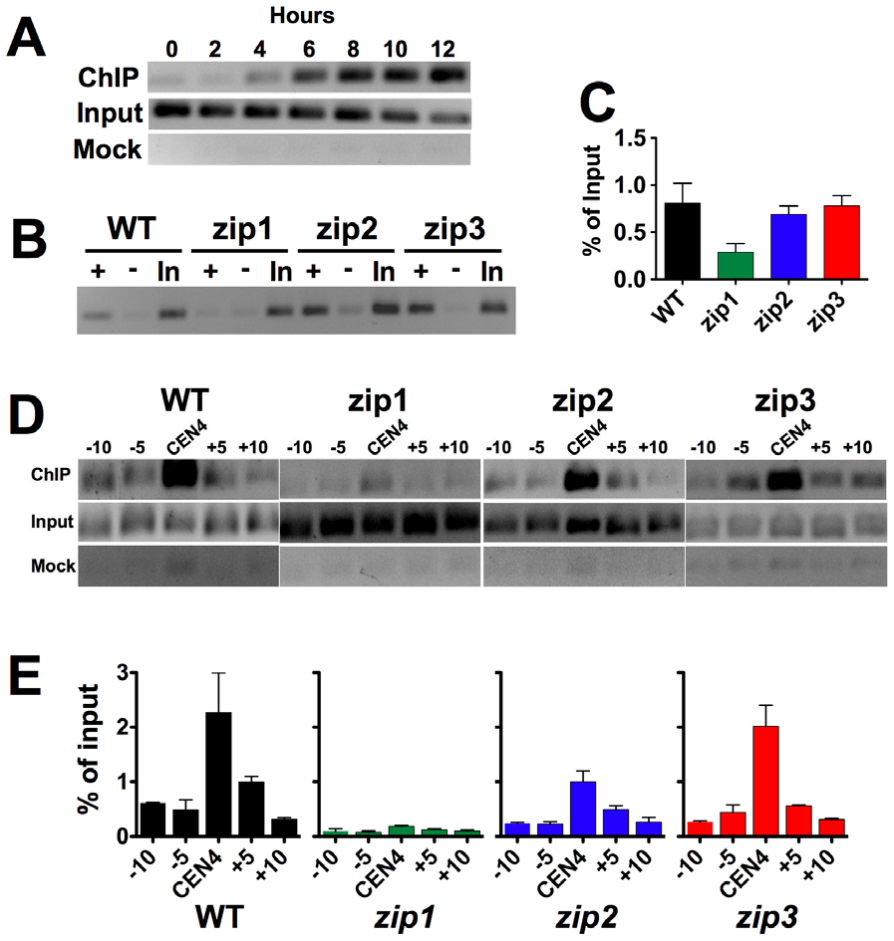
Zip1 associates with centromeres of non-exchange and exchange chromosomes in pachytene cells. (A) A wild-type yeast strain (DHC42) was induced to enter meiosis at 23°C, samples were harvested at timed intervals, and association of Zip1p with *CEN5* (from an exchange chromosome *V* pair) was evaluated with chromatin immuno-precipitation. PCR products were generated using primers spanning a ∼300 base pair region that includes *S. cerevisiae CEN5*. Input cell extract (In) diluted 1∶120, a mock immuno-precipitate in which no antibody was used (−) or the immuno-precipitate using antibody against Zip1p were used as templates in the PCR reaction. (B) Isogenic strains carrying a non-exchange chromosome *V* pair were induced to enter meiosis at 23°C (*WT*, DD728; *zip2*, DD732; *zip3*, DD737). Thirteen hours following meiotic induction samples were harvested for evaluating association of Zip1p with *CEN5* using chromatin immumunoprecipitation, as in (A), above. (C) Quantification of the ChIP PCR products shown in (A). The ratio of product in the (+) and (In) PCR reactions are shown. Values representing the average of three PCR reactions are shown. The error bars indicate SEM. (D) PCR products generated using primers that span ∼300 base pair regions at, and at 5 kb intervals extending outward from the centromeres (*CEN4*) of an exchange chromosome IV pair. (E) Quantification of the ChIP PCR products shown in (D).

The previous experiment provides qualitative evidence that Zip1 can be localized very close to the centromeres of non-exchange chromosomes in most wild-type nuclei, but doesn't offer the resolution to determine whether the Zip1 is at the core centromere region or associated with chromosome arms adjacent to centromeres. In *zip2*, *zip3*, and *zip4* mutants the centromeres of some non-exchange chromosomes are clearly associated with a clear Zip1 focus while others are not. It is difficult to conclude too much from these experiments due to the variability of the Zip1 signal intensity across the *zip2*, *zip3* and *zip4* spreads, the difficulty in identifying non-exchange chromosomes that are completely separated from all other chromatin, and the limitations in ascribing a precise chromosomal position to a Zip1 focus observed in a chromosome spread. To more precisely probe the association of Zip1 with the centromeres of non-exchange chromosomes in meiosis we turned to a chromatin immunoprecipitation (ChIP) protocol [26]. To test first whether Zip1 can be detected at the centromere regions of chromosomes with ChIP, we prepared extracts from meiotic cultures at a time point with maximal levels of pachytene cells. These were used in ChIP experiments with antibodies raised against Zip1. DNA isolated from immunoprecipitates was used as a template in PCR reactions to probe for the association of Zip1 with centromere regions. As a first test of this approach we probed Zip1 association with *CEN5* of exchange chromosomes in wild-type cells (Figure 4A). Zip1 association with *CEN5* could first be seen at four hours after the induction of meiosis and gradually increased as cells reached pachytene (12–13 hours post induction in this strain background). The assay was then used to monitor Zip1 association with the centromeres of non-exchange chromosomes in wild-type and *zip1*, *zip2* and *zip3* strains (Figure 4B and 4C). Zip1 was found to be associated with *CEN5*, and the association was independent of Zip2 and Zip3 (Figure 4B and 4C). In the same strains, Zip1 was also associated with the centromeres of exchange chromosomes (*CEN4*) in a manner independent of Zip2 and Zip3 (Figure 4D and 4E). To test whether this association was specific to the core centromere region we monitored the association of Zip1 at positions 5 and 10 kb on either side of the core centromere and found that Zip1 was especially enriched very close to the core centromere (Figure 4D and 4E).

### Zip1 mediates the association of homologous centromeres in meiosis I

**Figure 5:**
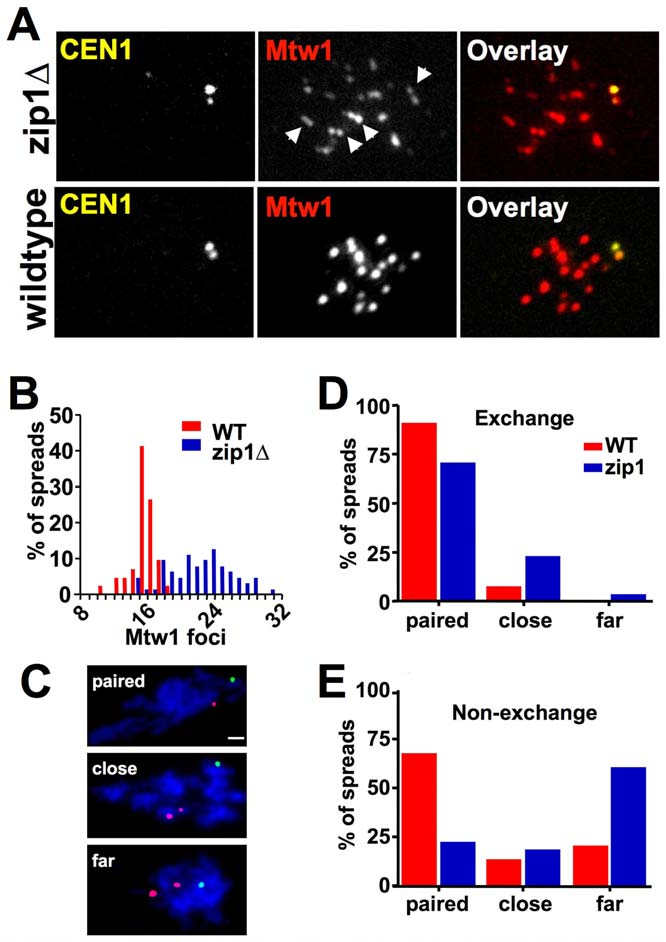
Zip1 promotes the association of exchange chromosome centromeres in pachytene. The association of homologous kinetochores was evaluated in chromosome spreads from wild-type and *zip1* strains. (A,B) Chromosome spreads were prepared from cells that expressed the epitope-tagged kinetochore protein Mtw1-13XMYC and carried a lacO/lacI-GFP tagged at both copies of *CEN1*. The number of Mtw1 foci was determined in cells with paired *CEN1*'s using indirect immunofluorescence microscopy. (A) shows representative wild-type and mutant spreads. Arrowheads indicate doublet Mtw1 foci common in *zip1* spreads. One of the doublets is the *CEN1* kinetochore pair, as this doublet Mtw1 signal co-localizes with the *CEN1* GFP tags. (B) shows quantification of Mtw1 foci (WT, DDO45, n = 41; *zip1*, DDO55, n = 31). (C,D) Association of homologous *CEN4* regions in wild-type (DMS383) and *zip1* (DMS384) strains. Both strains carried a *tet* operator array adjacent to *CEN4* and a *lac* operator locus on the arm of chromosome *VII*. Chromosome spreads were prepared for both strains. In spreads exhibiting condensed chromosomes and a single GFP dot, the distance between *CEN4* tetR-MYC foci was measured. Spreads were categorized according to the GFP inter-dot distance: paired (0–0.6 µm), close (0.8–2.0 µm), and far (>2.0 µm). (C) Examples of spreads in each category. The size bar equals 2 µm. (D) Quantification of *CEN4* separation (from an exchange chromosome *IV* pair) for *WT* (DMS383) and *zip1* (DMS384) diploids (n = 100 for each strain). (E) Quantification of separation of *CEN5* on non-exchange chromosomes in wild-type (DMS372) and *zip1* (DMS382) diploids (n = 100 for each strain).

Non-exchange chromosomes are relatively rare in budding yeast [15],[27],[28] raising the possibility that the centromere pairing process, observed using non-exchange chromosomes, has evolved mainly to mediate the behavior of exchange chromosome centromeres. The observation of pairing between the centromeres of non-exchange chromosomes is reasonably straight-forward: the pairing stands out because the arms of the non-exchange chromosomes are not aligned [12]. But this is not the case for exchange chromosome partners, which are aligned along their lengths in pachytene. Thus, in pachytene, the centromeres of homologous chromosomes are side-by-side, but whether they are actively paired cannot be determined by simple observation. If Zip1 acts to keep homologous (exchange) centromeres paired in pachytene, then in *zip1* mutants, the centromeres of homologous partners and their associated kinetochores, should be free to separate (within the constraints of flanking links such as chiasmata or SC associations, between the homologous partners). To test this we monitored the number of kinetochore foci in chromosome spreads of wild-type strains and *zip1* mutants. The gene encoding the kinetochore component, Mtw1, was modified to produce a functional, epitope-tagged Mtw1-13XMYC protein that could be used to detect the kinetochores in indirect immunofluorescence experiments. To allow the identification of chromosome spreads in which homologous chromosomes were paired, both copies of chromosome *I* were tagged with GFP at the centromere. Isogenic wild-type and *zip1* mutant versions of this strain were induced to enter meiosis and chromosome spreads were prepared (Figure 5A). Chromosome spreads with condensed chromosomes typical of late prophase, and with paired *CEN1's* (side-by-side or overlapping GFP foci) were then scored for the number of Mtw1-13XMYC (kinetochore) foci. The average number of Mtw1 foci increased significantly (unpaired t test, p<0.0001) from 16.0 (SD = 1.4) in wild-type spreads to 23.0 (SD = 4.4) in *zip1* spreads (Figure 5B). In the *zip1* spreads, we frequently observed doublet Mtw1-13XMYC foci that would be predicted if the kinetochores could separate slightly but remain tethered by flanking crossovers (Figure 5A, arrowheads). In cases where the GFP-tagged centromeres had separated, the two GFP-foci co-localized with the two foci of an Mtw1-13XMYC doublet (Figure 5A). This observation suggested that the observed increase in kinetochore foci might be due to an increase in slightly separated homologous centromere pairs in *zip1* strains. To test this notion, we assayed the pairing of a specific GFP-tagged pair of homologous centromeres (*CEN4*), in isogenic wild type and *zip1* mutant strains. These strains were modified to carry a *tet* operator array on the arm of both copies of chromosome *VII* (*AMS1*) and express a tetR-13XMYC gene fusion. Chromosome spreads were prepared from meiotic cultures of these strains. The chromosome spreads were first screened to identify those with condensed chromosomes and paired chromosome *VII* arms (one MYC dot). In these spreads we then measured the distance between the *CEN4*-GFP dots. The *CEN4*-GFP dots were categorized as being “paired” (0–0.6 µm), “close” (0.6–2.0 µm), or “far apart” (>2.0 µm). Representative spreads are shown in Figure 5C. The *zip1* mutation resulted in a significant increase in spreads with *CEN4*-GFP dots that were separated but close together (8.3% versus 23.7%; p<0.005). This is the predicted result if Zip1 normally links the centromeres, and if in the *zip1* mutant the centromeres can separate within the constraints of the flanking crossovers, and is similar to an observation made previously by Tsubouchi and colleagues [23],[24]. If the “close” centromeres in the *zip1* mutant are being prevented from further separation by crossovers, this would predict that in the absence of flanking crossovers to keep the centromeres close together, the loss of Zip1 would be predicted to cause an increase in the “far apart” category rather than the “close” category. We tested this by repeating the experiment, this time monitoring the behavior of GFP-tagged centromeres of a non-exchange chromosome pair (Figure 5E). In this case the “far” category increased significantly (from 20% to 60%, p<0.005) when Zip1 was absent.

### Zip1 is preferentially retained at centromeres upon synaptonemal complex disassembly

A model to explain the requirement for Zip1 for disjunction of non-exchange chromosomes is that Zip1 directly promotes a kinetochore organization that favors an initial attachment to microtubules that will direct the centromeres towards opposite poles of the spindle. The simplest form of this model is that Zip1 acts at centromeres until the time they attach to microtubules. The time at which centromeres attach to microtubules has not been fully established. By the time meiotic cells reach pachytene, the spindle pole bodies have been duplicated and lay side-by-side [29]. As cells exit pachytene the SC is disassembled and simultaneously the SPBs begin their separation to form a bipolar spindle [30],[31]. Electron microscopic examination of meiotic yeast cells suggested to Byers and colleagues that the first attachments of chromosomes to microtubules occur as the SC disassembles [31].

**Figure 6:**
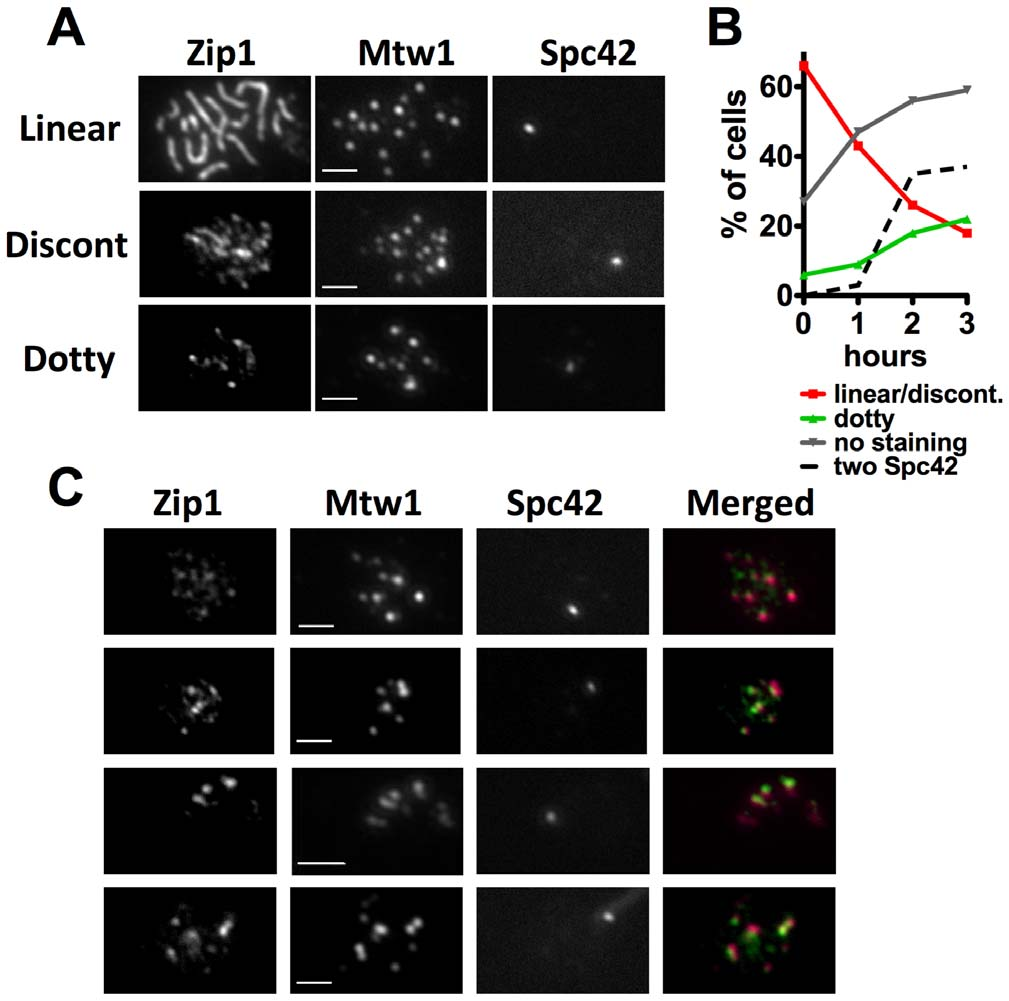
Zip1p is retained at centromeres during synaptonemal disassembly. The association of Zip1 with centromeres as cells exited pachytene was monitored using immunofluorescence microscopy. Cultures induced to undergo meiosis were arrested in pachytene by preventing expression of *P_GAL1_-NDT80* [32],[33]. Release from the pachytene arrest was triggered by addition of β-estradiol. Samples were harvest from the pachytene-arrested culture and at one-hour intervals following the release from pachytene. Chromosome spreads were prepared, and stained with anti-bodies against Zip1 and Mtw1-13XMYC. The natural fluorescence of Spc42-DSRed allowed visualization of spindle pole bodies. (A) Chromosome spreads were classified according to Zip1 staining as exhibiting linear, discontinuous (short linear segments and multiple dots of Zip1), or dotty (twenty or fewer Zip1 foci). (B) Proportion of spreads, by category, at pachytene arrest and at each time-point after release. The proportion of cells with separated Spc42-DSRed foci is also shown. n>61 for each time-point. (C) Co-localization of Zip1 foci and Mtw1-13XMYC was evaluated in chromosome spreads with twenty or fewer Zip1 foci (spreads from the two hour time point were used; n = 22 cells), all spreads in this category have one Spc42-DSRed focus.

To test whether Zip1 retains its association with centromeres until they become attached to microtubules we arrested cells in pachytene, released them from the arrest, and monitored Zip1 localization as cells progressed towards metaphase. To achieve this, we modified laboratory strains that exhibit a relatively rapid and efficient meiosis by placing the meiotic regulatory gene, *NDT80*, under the control of the *GAL1* promoter, and by introducing into the cells a construct that expresses a Gal4-estradiol receptor (Gal4-ER) hybrid protein [32],[33]. With this system, when cells are introduced into sporulation medium in the absence of β-estradiol the *NDT80* gene is not expressed. This results in a pachytene arrest with mature SC and duplicated spindle pole bodies [32]–[34]. At seven hours after induction of meiosis (pilot time courses demonstrated that most cells enter pachytene by six hours after induction), β-estradiol was added to the medium allowing Gal4-ER to induce *NDT80* expression, and a release from the pachytene arrest. Following the release from pachytene, cells were harvested at timed intervals, chromosome spreads were prepared and the staining patterns of Zip1 and its localization to kinetochores (Mtw1-13XMYC) was observed by indirect immunofluorescence. Chromosome spreads were categorized as having linear, discontinuous (many dots or short lines), dotty (fewer than twenty dots), or no Zip1 staining (see Figure 6A for representative spreads). At T = 0 (addition of estradiol), about 70% of the spreads exhibited Zip1 staining typical of pachytene (linear or discontinuous lines; Figure 6B, red). The proportion of cells with this staining pattern dropped rapidly after addition of β-estradiol. As cells with SC-like structures diminished those with small numbers of Zip1 foci (Figure 6B, green) and no Zip1 staining (Figure 6B, grey) appeared. The DS-Red tagged spindle pole body protein, Spc42-DS-Red, was used to monitor spindle morphology throughout the time course (Figure 6B, dashed black). The duplicated but unseparated SPBs of pachytene (Figure 6B, T = 0) were observed as a single DS-Red focus, which gave way to two foci as the SPBs separated to form a bipolar spindle (Figure 6B, T = 2).

The co-localization of Mtw1-13xMYC with Zip1 was used to explore the retention of Zip1 at centromeres as cells exited pachytene (examples in Figure 6C). We quantified the Mtw1/Zip1 co-localization in cells with fewer than twenty Zip1 foci (those in the final stages of disassembling the SC) at the T = 2 hour time point. These cells exhibited an average of 7.9 Mtw1 foci and 10.6 Zip1 foci (n = 175 Mtw1 foci, 233 Zip1 foci in 22 spreads). Mtw1 and Zip1 foci with a clear overlap were scored as co-localized. To test the significance of the observed co-localization, the level of co-localization in each chromosome spread was compared to the level observed when the Mtw1 and Zip1 foci were randomized by rotating the images 90° with respect to one another [35]. The values for observed versus randomized levels of co-localization were then compared with a Wilcoxon signed rank test. By this assay Mtw1 foci showed significant co-localization with Zip1 foci (59.4% observed versus 23.4% randomized, p<0.0001), and Zip1 foci showed significant co-localization with Mtw1 foci (44.6% observed versus 17.6% randomized, p<0.0001).

**Figure 7:**
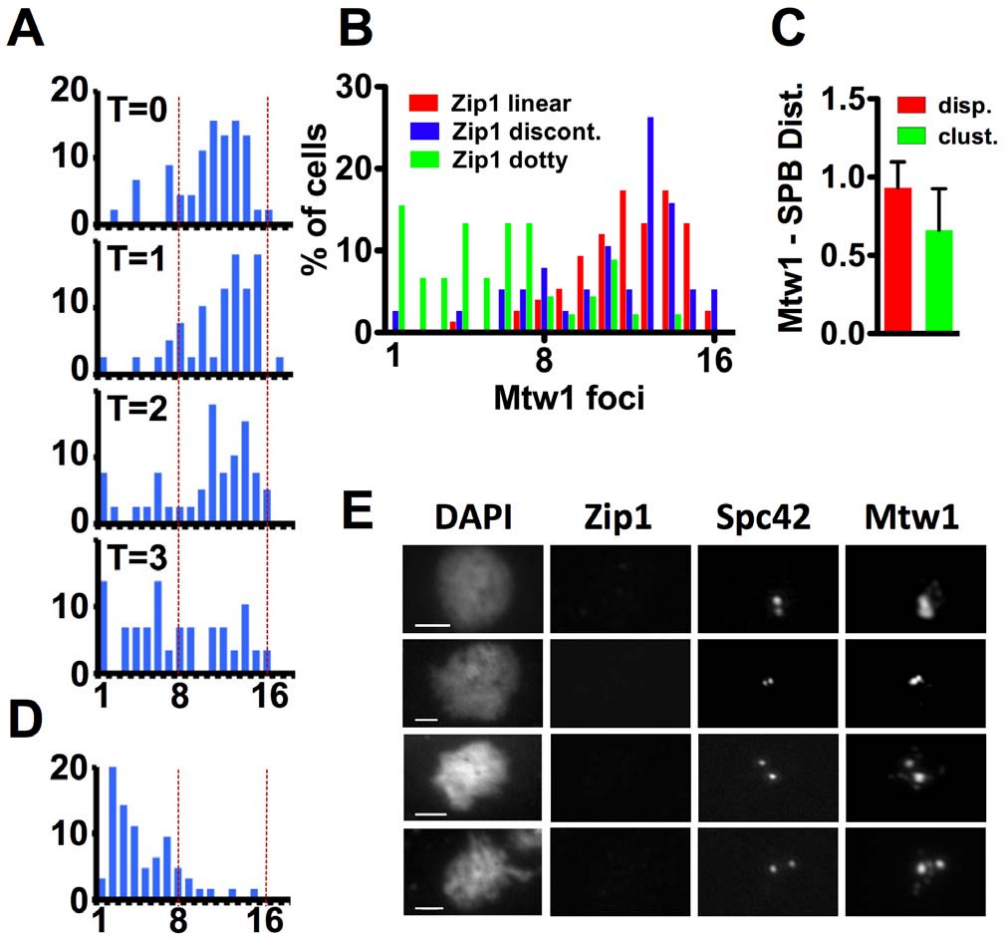
Zip1 persists at centromeres as they migrate to spindle poles and disappears concomitant with spindle separation. (A) Number of Mtw1-13XMYC foci in chromosome spreads from pachytene arrested cells and at each time point after release from the arrest. Only cells with positive Zip1 staining and one Spc42-DSRed focus are included (n>29 for each time point). (B) Number of Mtw1 foci in chromosome spreads classified according to Zip1 morphology (taken from Zip1 positive spreads at all time points in the time course; n = 158). (C) Kinetochore-to-SPB interval size in chromosome spreads with clustered or non-clustered kinetochores. The distance between each Mtw1 focus and the SPB was determined for chromosome spreads with clustered (dotty Zip1 and fewer than twelve Mtw1 foci) or dispersed (linear Zip1 and greater than twelve Mtw1 foci) Mtw1 foci to evaluate whether clustering of Mtw1 foci is correlated with movement towards the SPB. The average Mtw1-SPB interval was determined for each chromosome spread then normalized for the area of the spread (see Materials and Methods for details). Shown are the average, normalized Mtw1-SPB interval lengths of twenty five spreads with dispersed (red) or clustered (green) kinetochores. Error bars represent one standard deviation. (D) Number of Mtw1-13XMYC foci in chromosomal spreads with two Spc42-DSRed foci (n = 63). (D) Examples of chromosomal spreads of cells with two close Spc42-DSRed foci.

The chromosome spreads from the pachytene arrested cells (Figure 6A, linear) usually featured about sixteen Mtw1 foci, one for each chromosome pair, while chromosome spreads with small numbers of Zip1 foci (dotty) that appeared as the cells exited from pachytene often had many fewer than sixteen Mtw1 foci (Figure 6A, dotty). The number of Mtw1 foci in cells harvested at time points after the release from pachytene was quantified (Figure 7A). Most Zip1-positive chromosome spreads from the arrested cells (T = 0) exhibited a number of Mtw1 foci close to the sixteen expected if each bivalent yields a single focus, consistent with previous observations [13],[36] (Figure 7A, T = 0). Following release from the pachytene arrest, a prominent population of cells emerged with fewer Mtw1 foci (Figure 7A, T = 3). To determine whether the reduced number of Mtw1 foci was correlated with Zip1 status, we determined the number of Mtw1 foci in cells scored as having linear, discontinuous, or dotty Zip1 staining (Figure 7B). The average number of Mtw1 foci dropped slightly as cells proceeded from linear to discontinuous SC (from 12.2 foci/spread, SD 2.4, to 11.3 foci/spread, SD 3.4) then dropped to a significantly lower number in cells with dotty Zip1 (5.6 foci/spread, SD 3.5, unpaired t test, p<0.0001), consistent with a clustering of the kinetochores concomitant with SC disassembly. In most dotty spreads (Figure 6C), one or a few of the Mtw1 foci overlapped, or were immediately adjacent to, the single Spc42 focus. Therefore as the clustering of Mtw1 signals is taking place, some of the kinetochores are becoming associated with the side-by-side SPBs. To test whether the clustering of the kinetochores indicates directed movement towards the SPBs as opposed to aggregation of the kinetochores independent of directed movement towards to SPBs, we tested whether the average Mtw1-to-SPBs interval size is reduced as the centromeres cluster. The distance between each kinetochore (Mtw1) focus and the SPBs was measured in chromosome spreads with clustered (fewer than twelve) or dispersed Mtw1 (more than twelve) foci from the “dotty” and “linear” chromosome spreads respectively in Figure 7B, and the average Mtw1-to-SPBs distance, normalized for the size of the spread, was determined. In chromosome spreads with clustered Mtw1 foci, the foci were significantly closer to the SPBs than was true for spreads with dispersed foci (unpaired t test, p<0.0001) (Figure 7C). Thus the clustering of kinetochores in these spreads coincides with movement towards the SPBs.

To determine whether the centromere-associated Zip1 persists into metaphase we evaluated Zip1 staining in cells that had developed bipolar spindles – those with two Spc42 foci (Figure 7D and 7E). In these chromosome spreads, Zip1 was rarely detectable even with long exposures (Figure 7E). As a second test of the persistence of Zip1 at centromeres into metaphase, meiotic cells were arrested at metaphase by down-regulating expression of *CDC20*. Here too, Zip1 was not detected in metaphase cells (not shown). In spreads with separated spindle pole bodies Mtw1 was always found as one focus, or a few tightly bunched foci, at each pole (Figure 7D and 7E). Thus, the kinetochores have migrated to the poles prior to the time that discernable separation of the spindle pole bodies has occurred.

### Zip1 promotes proper spindle orientation of homologous chromosomes in meiosis I

**Figure 8:**
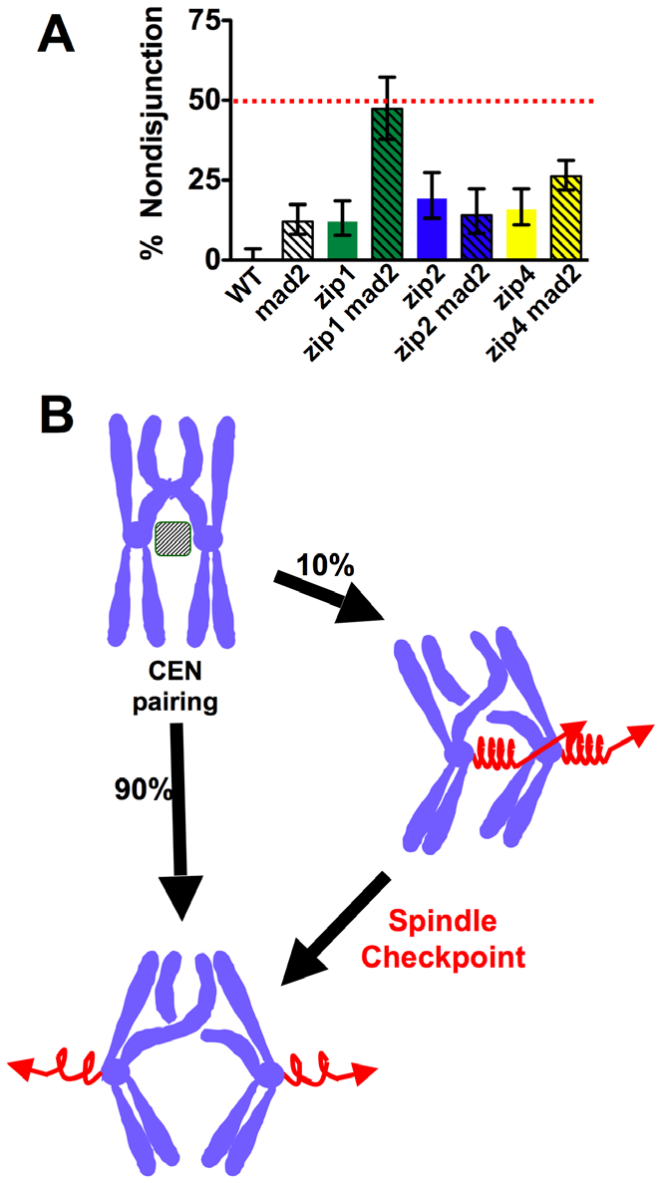
Zip1p promotes proper orientation of homologous chromosomes at meiosis I. Isogenic strains bearing chromosomes with a GFP tag adjacent to CEN4 were induced to enter meiosis. Samples were briefly fixed, stained with DAPI then visualized using fluorescence microscopy to monitor the distribution of GFP foci in bi-nucleate cells. (A) Nondisjunction frequencies were determined for homologous chromosome *V*'s in *WT* (DMS371; n = 112), *zip1Δ* (DMS381; n = 100), *mad2Δ* (DMS387; n = 98). The dashed black line represents random chromosome segregation (50% nondisjunction). (B) A model for the mechanism by which centromere pairing and the spindle checkpoint act together to ensure proper chromosome segregation in meiosis I.

The finding that Zip1 promotes pairing of exchange chromosome centromeres raised the question of whether this pairing promotes the formation of bipolar spindle attachments for exchange chromosome partners as it does for non-exchange chromosome partners. In budding yeast, the spindle checkpoint provides a short metaphase delay when the cell is faced with chromosomes that have not achieved a stable bipolar spindle attachment [3],[37]. After this delay, the cells proceed with anaphase I, even if the improper attachments have not been rectified [3],[37]. If Zip1 acts to ensure that most initial microtubule attachments of exchange bivalents will be of a bipolar orientation, then loss of Zip1 should result in an increase of improperly attached bivalents and an increased need for the spindle checkpoint. We tested this prediction by monitoring the segregation of an exchange chromosome pair in cells deleted for the spindle checkpoint gene, *MAD2*, in *zip1* mutants, and in *zip1 mad2* double mutants (Figure 8A). In the absence of *mad2*, chromosome *IV* exhibited about 13% non-disjunction, similar to previously reported values [37], suggesting that in about 90% of meioses chromosome *IV* does not require a spindle checkpoint delay or the re-orientation activity of Mad2 to achieve a bipolar spindle attachment [38]. This 90% level of correct spindle attachment exhibited by chromosome *IV* in a *mad2* mutant background is similar to the segregation fidelity of a non-exchange chromosome pair in wild-type cells and suggests that for both exchange and non-exchange chromosomes, in about 90% of meioses, the initial attachment of the centromeres to the spindle is in a bipolar configuration. In *zip1* mutants (with a functional spindle checkpoint) we observed about 10% non-disjunction. This result suggests the possibility that bivalents in *zip1* mutants experience elevated levels of improper microtubule attachments and the presence of a functional spindle checkpoint allows all but 10% of the improper attachments to be corrected. By this model, the double *zip1 mad2* mutant should show very high levels of non-disjunction and this is the case (49% non-disjunction, Figure 8A). To test whether the *zip1* defect in bi-orientation is due to a centromere-pairing defect or instead a defect in SC formation the same experiment was performed in *zip2* and *zip4* mutants. Previous work has demonstrated that *zip4* mutants have defective SC assembly but functional Zip1-dependent pairing of exchange chromosome centromeres [24]. Both single mutants have slight increases in non-disjunction but unlike *zip1* mutants do not have synthetic defects with *mad2* (Figure 8A), consistent with the model that a major component of the segregation defects of *zip1* mutants are attributable to failures in centromere pairing.

## Discussion

### Zip1 is necessary for the pairing and segregation of non-exchange chromosomes

It has been known for some years that yeast has a mechanism that enhances the segregation fidelity of chromosome pairs that have failed to experience exchange [39]. Subsequent studies demonstrated that the non-exchange disjunction process in budding yeast, like that in other model organisms, includes a period of centromere pairing that persists until just before the non-exchange partners disjoin on the metaphase spindle [12]. The experiments presented here begin to provide clues to the molecular basis of this centromere pairing. The synaptonemal complex component, Zip1 is shown here to be required for both the pairing of centromeres of non-exchange chromosomes, and for their disjunction. This result is in contrast to our earlier observations of the role of Zip1 in non-exchange segregation behavior using tetrad dissection. In that study we saw no large impact on non-exchange disjunction when *ZIP1* was deleted [12]. The explanation is that in *zip1* mutants, meioses in which the test chromosome non-disjoined did not produce tetrads as efficiently as those with proper segregation, resulting in an under-representation of non-disjunction tetrads. Similar observations have been reported by others [27]. Unlike the tetrad dissection approach, the approach used here, the observation of chromosomes in meiotic cells, directly measures the anaphase outcomes and is not biased by the viability of the meiotic products.

Two other proteins that are required for organization of Zip1 into a synaptonemal complex, Zip2 and Zip3, were also found to be important for centromere pairing and non-exchange chromosome disjunction. These proteins are not required for the period of homology-independent pairing of centromeres that occurs in early prophase ([13] and unpublished observations), nor are they required for Zip1 to associate with centromeres according to our ChIP assays. We do not know how the centromeric association of Zip1 detected by ChIP corresponds to the Zip1 foci observed in immunofluorescence experiments. It may be that the ChIP approach identifies the centromeric association of small numbers of Zip1 molecules that might not produce the striking Zip1 foci seen by fluorescence microscopy.

If Zip1 is able to localize to the centromeres in *zip2* and *zip3* mutants, then why do these mutants exhibit a defect in centromere pairing of the non-exchange chromosomes? One possibility is that Zip2 and Zip3 are required for the transition from the early stage of homology-independent centromere pairing (before pachytene) to the alignment of centromeres with their homologous partners and the initiation of synapsis as cells move towards pachytene. By this model, in *zip2* and *zip3* mutants, the centromeres of the non-exchange chromosome *V*'s (and all the other chromosomes) pair with random partners in early prophase, but are unable to release from those partners as cells proceed into pachytene. Indeed, Zip3 has recently been shown to block the initiation of synapsis at centromeres in the absence of recombination [40]. In *zip3* mutants, precocious SC formation between non-homologous centromere pairs in early meiosis could be imagined to lock the centromeres of the non-exchange chromosomes to their early prophase pairing partners, possibly preventing the non-exchange chromosome centromeres from aligning with one another in pachytene.

The finding that Zip1 is at the centromeres of non-exchange chromosomes and mediates their disjunction raises the question of the nature of the structure that Zip1 forms at the centromeres. Electron microscopy was used previously to examine chromosome spreads from a strain carrying one non-exchange chromosome pair [41]. This work suggested that non-exchange partners assemble axial elements, but instead of being arranged in parallel, with a bridging central element, they were disordered with one or more sites of apparent contact [41]. How the axial elements of the non-exchange partners were linked was not clear, but our demonstration of a requirement for Zip1 in the pairing of non-exchange partners suggests that Zip1 might promote the association of the non-exchange chromosome cores.

### Centromere pairing and microtubule attachment

The centromere pairing of non-exchange partners in late meiotic prophase promotes their disjunction [12]. Unlike exchange chromosomes, which separate at anaphase I, non-exchange partners lose their association at metaphase I suggesting that meiotic centromere pairing is lost by metaphase or overcome upon the application of poleward forces on the partner chromosomes [12]. Consistent with this, at metaphase we did not detect any Zip 1 associated with the chromosomes suggesting that by that stage most Zip1 has been released from the centromeres. These observations suggested the model that the primary contribution of centromere pairing to the segregation of non-exchange chromosome partners is to optimize the chance that initial microtubule attachments will be in the bipolar configuration [6],[7],[12].

Our observations of the timing of Zip1 removal from the chromosomes demonstrate that Zip1 is preferentially retained at centromeres when the SC is disassembled and remains associated until the time centromeres are becoming attached to microtubules. As the SC is being disassembled, the numbers of kinetochore foci are considerably reduced suggesting an aggregation of the centromeres. This aggregation could in part be due to centromere-centromere associations (which have been reported in late meiotic prophase in many organisms, [7]) but the fact that the kinetochores are moving closer to the SPBs suggests that some of the aggregation is by concentration at the SPBs. These findings are congruent with early observations of meiotic progression using electron microscopy [31]. These studies led to the conclusion that microtubules, emanating from side-by-side spindle pole bodies, begin establishing contacts with the chromosomes as cells exit pachytene.

By the time spindle poles separate to yield even very short bipolar spindles, the centromeres are concentrated at the two poles. Thus, most attachment of kinetochores to microtubules has occurred before the spindle pole bodies separate sufficiently to yield two foci (greater than 0.5 µm) in our fluorescence microscopy assays. Zip1 remains at centromeres as cells are in the process of forming kinetochore-microtubule attachments, and loss of most Zip1 from the centromeres occurs concomitant with, or just prior to, the formation of clear bi-polar spindles. The signal for the removal of the final centromere-proximal Zip1 remains unsolved. Zip1 could be released from centromeres as each one attaches to a centromere or the release could be global, as cells progress towards metaphase.

### Zip1 mediates pairing of exchange chromosome centromeres

Though most easily evaluated with non-exchange chromosomes, the greater impact of Zip1-mediated centromere pairing likely lies in the behavior of exchange chromosomes. In the absence of Zip1, the centromeres of exchange chromosomes are often separated in chromosome spreads consistent with the notion that Zip1 can actively pair the centromeres of exchange chromosomes just as it can with non-exchange chromosomes. Recent observations by Tsubouchi and colleagues (2008) have led the to the similar conclusion that Zip1 acts to promote the association of the centromeres of exchange chromosomes, and this pairing activity can occur independently of SC assembly. This centromere pairing appears to promote proper bipolar spindle attachments of exchange chromosome pairs. The fact that the non-disjunction frequency of exchange chromosome pairs is only about 10% in spindle checkpoint (*mad1* and *mad2*) mutants [12],[37] demonstrates that alternative mechanisms beyond the spindle checkpoint can promote the establishment of a bipolar attachment of homologous centromeres to the meiotic spindle. The random segregation of exchange chromosomes in the *zip1 mad2* mutants reveals that Zip1 is a necessary component of at least one such alternate mechanism. These results suggest that the spindle checkpoint and a centromere orientation mechanism that includes Zip1 provide redundant opportunities for achieving bipolar spindle attachment (Figure 8B). By this model, Zip1 acts to promote the initial attachment of microtubules to partner centromeres in the correct, bipolar, configuration for about 90% of chromosome pairs (the frequency of proper attachment for non-exchange pairs that presumably cannot benefit from a spindle checkpoint dependent re-orientation process). Those few bivalents that are improperly attached to microtubules then depend upon the spindle checkpoint for correction. These two steps probably occur only in this order. When the centromeres are first attached to the microtubules, the SPBs are side-by-side. It is probably not until the SPBs fully separate to produce a metaphase spindle that there is a sufficient interpolar distance to generate tension at the kinetochores of properly attached bivalents, thus stabilizing kinetochore microtubule attachments [42].

Whether centromere pairing is a widely conserved mechanism for promoting disjunction of exchange chromosomes is unclear. Early descriptions of meiosis in mouse spermatocytes revealed that homologous centromeres remain associated after SC disassembles [43],[44]. While the SC central element cognate of Zip1 (SYCP1) is removed from the mouse chromosomes as SC disassembles, a lateral element component (SYCP3) persists at the paired centromeres [45]. Whether SYCP3, like Zip1, is contributing to bipolar attachment of the centromeres in mouse spermatocytes is not known. What is clear, primarily from studies of the segregation of achiasmate chromosomes from a wide array of model organisms, is that the recruitment of components of the SC to provide either linkage or centromere orientation of chromosome partners is a recurring theme in meiotic biology (for examples see, [46]–[48]; reviewed in [49],[50]).

Any process that reduces the workload of the spindle checkpoint could have significant implications for human chromosome segregation fidelity. Mammalian oocytes (like budding yeast) are prone to experience short, often insufficient, checkpoint delays when faced with errant chromosomes [4],[51]. This results in chromosome mis-segregation, and aneuploid gametes, when inappropriate spindle attachments are not rectified before the checkpoint releases cells into anaphase I. Redundant mechanisms, like centromere pairing, that reduce the workload for the spindle checkpoint could be significant contributors to meiotic segregation fidelity, and defects in these processes could result in the production of aneuploid gametes.

## Materials and Methods

### Yeast strains and culture conditions

Strains are S288C derivatives [52] or (DDO diploids) are isogenic derivatives of rapidly sporulating strains, of primarily S288C and W303 ancestry, derived in the RE Esposito laboratory [53]. Genotypes are shown in Table S1. We used standard yeast media and culture [54]. To induce meiosis, cells were grown in YP-acetate to 3–4×10^7^ cells per ml, and then shifted to 1% potassium acetate at 10^8^ cells per ml.

### Strain construction

Genetic methods were performed according to standard protocols [54]. PCR-based methods were used to create complete deletions of ORFs and epitope-tags [55]. Some deletions were created by using PCR to amplify deletion-KANMX insertions from the yeast gene deletion collection (Invitrogen) and these products were then used for transformations. Diagnostic PCRs were performed to confirm each gene modification.

### Meiotic chromosome spread preparation and analysis

Meiotic nuclear spreads were prepared from cells cultured at 30°C according to published protocols with the following modifications [30]. Cells were spheroplasted using 20 mg per ml zymolyase 100T for approximately 30 minutes. Spheroplasts were briefly suspended in MEM (100mM MES, 10mM EDTA, 500uM MgCl_2_) containing 1mM PMSF, fixed with 4% paraformaldehyde and spread onto poly-L lysine- coated slides (Fisherbrand Superfrost Plus). Slides were blocked with 4% non-fat dry milk in phosphate buffered saline for at least 30 minutes, and incubated overnight at 4°C with primary antibodies. Primary antibodies were mouse anti-Zip1p (a gift from Rebecca Maxfield), rabbit anti-MYC (Bethyl Laboratories A190-105A), mouse anti-MYC, (gift from S. Rankin), chicken anti-GFP (Chemicon AB16901), and rabbit anti-GFP (Invitrogen A11122). Secondary antibodies were Alexa Fluor 488-conjugated goat anti-chicken IgG, Alexa Fluor 546-conjugated goat anti-mouse IgG, and Alexa Fluor 647 conjugated goat anti-rabbit IgG, Alexa Fluor 568-conjugated anti mouse (all from Molecular Probes). Secondary antibody incubations were for two hours at room temperature. Control experiments in which individual primary antibodies (anti-Zip1 and anti-MYC) were omitted revealed that signals obtained with when evaluating Zip1 and GFP localization were restricted to their assigned channels and gave no detectable “bleed-through” into other channels using the exposure settings employed for these experiments.

Centromere pairing in pachytene was evaluated using published methods [12] in which a *lac* operator array was inserted adjacent to the centromere of two chromosomes and a lacI-GFP hybrid protein was expressed under the control of a meiotic promoter to produce a focus of GFP at the *lac* operator arrays. Chromosome spreads were prepared and indirect immunofluorescence was used to identify those spreads with the condensed chromosomes typical of late meiotic prophase and the number of GFP and proximity of foci was used as a measure of pairing. Spreads with one focus or two foci within 0.6 microns were scored as paired. Those in which the foci were separated by a larger distance were scored as unpaired. To determine the frequency with which spreads with a single GFP focus might be due to failure to detect on focus (*e.g.* loss of the lac operator array, failure of a chromosomes to stick to the slide or a weak immunofluorescence signal), we prepared and scored chromosome spreads from cells in which only one chromosome of a non-exchange chromosome pair carried a *lac* operator array (in both wild-type and zip1 strain backgrounds). Chromosome spreads that fail (for any reason) to give a detectable signal from the one lac operator tagged chromosome would have no GFP focus. In this assay, wild-type cells (DD770) 5.0% of cells had no signal (n = 100) and in *zip1* cells (DHC54) 5.4% of the cells had no signal. There appears to be no Zip1-dependent affect on loss of the GFP signal, but the pairing values (spreads with one GFP focus) in our assays are probably a slight over estimate.

For statistical studies, probabilities were determined for 2×2 contingency tables using the summing small P values method of Fisher's exact test (two sided), or where indicated by Wilcoxon tests of significance.

### Analysis of meiosis I non-disjunction

Non-disjunction frequencies of homologous or homeologous (non-exchange) chromosome pairs were determined using published methods [12], in which strains were modified so that one chromosome pair carried an array of *lac* operator repeats near the centromere and expressed a lacI-GFP fusion protein that would bind to the array producing a green GFP focus at that position. These strains were induced to enter meiosis at 23°C (because *zip1*, *zip2* and *zip3* mutants in this strain background arrest in pachytene at 30°C) and cells were harvested at time determined in pilot experiments to correspond to anaphase I. Cells were fixed for 5 minutes at room temperature in 5% formaldahyde, washed one time with PBS and then stored at 4°C. These cells were stained with DAPI and observed using fluorescence microscopy. Cells with one GFP focus in each DAPI mass of an anaphase I cell were scored as having experienced disjunction, those with one or two GFP foci confined to one of the DAPI masses of an anaphase I cell were scored as having experienced non-disjunction. Non-disjunction was also evaluated in tetranucleate cells (GFP foci restricted to two DAPI masses scored as non-disjunction, in all four DAPI masses scored as disjunction). Values obtained in tetranucleate cells corresponded closely to those obtained in binucleate (anaphase I) cells (not shown).

To determine to rate at which anaphase cells with a single GFP focus resulted not from non-disjunction, but from the failure to detect the GFP signal from one of the chromosomes (*e.g.* thru loss of the lac operator array, the entire chromosome, or a weak GFP signal), cells with only a single chromosome tagged with GFP were analysed as described. We evaluated strains in which one chromosome of a non-exchange chromosome pair was tagged in both wild-type (DD770) and *zip1* strain (DHC54) backgrounds. Loss of a detectable GFP focus would yield an anaphase cell with no GFP focus. Wild-type cells yielded 3% loss of the signal (n = 66) and the *zip1* strain yielded 0% loss (n = 50), suggesting that loss of the GFP signal has a very small impact on measured non-disjunction frequency.

### Generation and analysis of synchronous post-pachytene cultures

To examine post-pachytene cells we eliminated the asynchrony caused by the variation in timing of entry into the meiotic program by reversibly arresting cells in pachytene [32],[33]. PCR-based methods were used to develop laboratory strains with the meiotic regulatory gene, *NDT80*, under the control of the *GAL1* promoter. A construct that expresses a Gal4-estradiol receptor (Gal4-ER) hybrid protein (a gift from K. Benjamin) was stably introduced into these cells. *P_GAL1_- NDT80*, Gal4-ER strains were confirmed to arrest in pachytene and resume meiosis after the addition of 7µM β-estradiol to the culture medium. Cells were added to sporulation medium in the absence of β-estradiol. At 7 or 9 hours, 7µM β-estradiol was added (T = 0), samples were taken each hour after, and chromosomal spread analysis was performed as described above.

### Measuring relative distances from the kinetochores to the SPBs in chromosome spreads

To determine whether the observed clustering of kinetochores in chromosome spreads from cells exiting pachytene coincided with movement towards the SPBs we determined the average distance between Mtw1-13XMYC foci and the SPB in nuclei from different classes. Because the radii of the chromosome spreads are variable the average Mtw1 to SPB distance for each spread was normalized by dividing by a factor that reflected the size of the spread. The following methods were used. Measurements were determined for twenty five nuclei with clustered Mtw1 foci (dotty Zip1 staining and fewer than 12 Mtw1 foci) and for twenty five nuclei with dispersed Mtw1 foci (linear Zip1 and over 12 Mtw1 foci). For each spread the distance from each Mtw1 focus to the SPB was determined (using Axiovision software) these Mtw1-SPB interval lengths were averaged. Next, the area covered by the spread was determined (using Axiovision software). The radius that would give a circle of that area was then calculated. The average Mtw1-SPB distance for each chromosome spread was then normalized to the size of the spread by dividing by the radius. The normalized Mtw1-SPB distances for the twenty five chromosome spreads in each category were averaged and the standard deviation determined. Note that for the clustered chromosome spreads, some Mtw1 foci contain multiple kinetochores yet the Mtw1-SPB interval for these foci is not weighted to represent this. If clusters are more likely to be near the SPB, then the approach under-estimates the bias for proximity of kinetochores to the SPB in chromosome spreads with clustered kinetochores.

### Microscopy

Images were collected using a Zeiss AxioImager microscope with band-pass emission filters, a Roper HQ2 CCD, and Axiovision software. Inter-GFP dot distances were determined using Axiovision software.

### Chromatin immunoprecipitation (ChIP)

ChIP was performed according to [26] with minor modifications. Approximately 2×10^8^ cells were used per ChIP experiment (mock, IP and input). Chromatin was formaldehyde-crosslinked for 30 minutes at room temperature and sonicated to obtain average fragment sizes of 500–700 bp. Antibodies used for ChIP was rabbit polyclonal anti-Zip1p (Santa Cruz Biotechnology, sc-33733). Protein-G sepharose beads were from Invitrogen. After reversal of cross-linking, overnight at 65°C, DNA was purified using QIAquick PCR purification kit (Qiagen) according to the manufacturer's instructions. PCR was used to amplify selected chromosomal regions. Primers were chosen to amplify ∼300 bp fragments. Primer sequences and co-ordinates are listed in Table S2. The number of PCR cycles to be used for each primer was determined empirically so as not to reach saturation. Input DNA was diluted 120 times. PCRs were performed with Denville Hot-Start Taq DNA Polymerase. 25–30 ul PCR reactions were run on a 1.2% agarose gels. Images were obtained with a Kodak Image Station 4000R. Band intensities were measured using Kodak Molecular Imaging Software version 4. Each ChIP experiment was performed two or more times with similar outcomes and a representative experiment is shown. Two or more PCR analyses were performed for each ChIP that is presented. Error bars reflect the variation (standard error of the mean) among these PCRs.

## Supporting Information

Table S1 Strains used in this study.
(0.08 MB DOC)

Table S2 Primers used for Chromatin Immuno-precipitation.
(0.04 MB DOC)
