# Supplementary material for: The Synaptonemal Complex Protein Zip1 Promotes Bi-Orientation of Centromeres at Meiosis I
Source: PLoS Genet. 2009 Dec 11;5(12):e1000771. doi: 10.1371/journal.pgen.1000771 (PMC2781170; doi:10.1371/journal.pgen.1000771)
Supplement: Table S1 — Strains used in this study. (0.08 MB DOC) [file pgen.1000771.s001.doc]

**Table S1.** Strains used in this study

| Strain | Genotype |
| --- | --- |
| DMS127-1.6A | *MAT****α*** *, ade1::ARG, leu2, trp1 or trp2, ndc10-1,*  *S. cerevisiae* chromosome *V*:  *ura3::HIS3::[pAFS152:URA3 P_CYC_-GFP-lacI], sec3:: [pBK13.1-LEU2-lacO]* |
| DMS143.16A | *MAT***a***, his3-1, 15, lys2-801::[pLL1.1: LYS2 P_CYCl_ lacI-GFP], arg4-ΔHpa, S. carlbergensis* chromosome *V: URA3, pac2::[pD174:LEU2 lacO], ilv1* |
| DMS175.20A | *MAT***a***, his3, lys2-801::[pLL1.1:LYS2 P_CYC_lacI-GFP], , ndc10-1*  *S. carlsbergensis* chromosome *V*: *URA3, pac2::[pD174: LEU2 lacO]* |
| DMS301.12D | *MAT, ura3-52::[pAFS152-lacIGFP-URA3], trp1-289, zip1::KAN* |
| DMS320.14B | *MAT***a***, ura3::HIS3, his3-11,15, lys2-801::[pLL1.1:LYS2-P_CYC1_-GFP-lacI], sec3:: [pBK13.1:LEU lacO], zip1::KAN* |
| DMS347.2A | *MAT***a***, ade2, cyh2, his7-1, leu2, lys2-2, met13-c, trp1-63::[pMNS22:tetRMYC TRP KAN], tyr1-2, ura3-13::[pAFS152:URA3 Pr_CYC_-lacI-GFP], CEN4::[pMNS23: tetO URA3], AMS1::[lacO-LEU2], zip1::KANMX* |
| DMS348.42A | *MAT********, ade2, lys2-2::[P_DMC1_-lacI-GFP-LYS2], his7-1, met13-c, trp1Δ63::[pMNS22:tetR-MYC-G418-TRP1], tyr1-2, ura3-1::[P_CYC1_-lacI-GFP URA3], cyh2 , AMS1::[lacO LEU2], CEN4::[pMNS23:tetO-URA3]* |
| DMS350.11D | *MAT* **a***, ura3::HIS3, his3-11,15, lys2-801::[pLL1.1: LYS2 Pr_CYC_GFP lacI], sec3::[pBK13.1:LEU lacO]* |
| DMS351.4A | *MAT***a***, leu2, lys2-801::[pLL1.1: LYS2 Pr_CYC_GFP lacI], pac2::[pD174:LEU2 lacO], pch2::URA3, URA3, ?rad3?, arg4, cup1::ura3::THR?, his3, zip1::KAN* |
| DMS377.18A | *MAT***a***, leu2, ade5, can1, lys2-1, met13-d, trp1-Δ63, tyr1-1, ura3-13, his7, AMS1::[lacO-LEU2], CYC1::[P_CYC1_lacI-GFP URA3], CEN4::[pMNS23:tetO URA3]* |
| DMS377.29B | *MAT***a***, leu2, ade5, can1, lys2-1, met13-d, trp1-Δ63, tyr1-1, ura3-13::[pAFS152:URA3 Pr_CYC_-lacI-GFP], his7, AMS1::[lacO-LEU2], CYC1::[P_CYC1_lacI-GFP URA3], CEN4::[pMNS23: tetO URA3], zip1::KANMX* |
| MDY2196 | *MAT********, lys2-2::[P_DMC1_-lacI-GFP LYS2], URA3::[P_CYC1_-lacI-GFP], AMS1::[lacO LEU2], ade2, leu2, lys2-2, his7-1, met13-c, trp1-63, tyr1-2, ura3-1, cyh2* |
| TMS46-7.3D | *MAT****α****, ade1::ARG4, trp2, leu2, his3-11,15, arg4-ΔHpa, lys1,*  *S. cerevisiae* chromosome *V: rad3, ilv1, ura3::HIS3::[pAFS152:URA3 Pr_CYC_- lacI-GFP], sec3::[pBK13.1:LEU2 lacO]* |
| TMS187 | *MAT********, lys2-2::[P_DMC1_-lacI-GFP-LYS2], ura3::[P_CYC1_-lacI-GFP URA3], AMS1::[lacO LEU2], ade2, leu2, lys2-2, his7-1, met13-c, trp1-63, tyr1-2, ura3-1, cyh2, zip1::KANMX* |
| TMS189 | *MAT***a***, URA3::[P_DMC1_-lacI-GFP URA3], CYC1::[P_CYC1_lacI-GFP URA3], AMS1::[lacO LEU2], ade5, can1, his7-2, leu1,2, lys2-1, met13-d, trp1-63, tyr1, ura3-13, MTW1-13XMYC-TRP1* |

| TMS196 | *MAT***a***, URA3::[P_DMC1_-lacI-GFP URA3], CYC1::[P_CYC1_lacI-GFP URA3], AMS1::[lacO LEU2], ade5, can1, his7-2, leu1,2, lys2-1, met13-d, trp1-63, tyr1, ura3-13, MTW1-13XMYC-TRP1, zip1::KANMX* |
| --- | --- |
| TMS199 | *MAT****α****, ade1::ARG4 ,his3-11,15, arg4-ΔHpa , zip1::G418*  *S. cerevisiae* chromosome *V: rad3, ilv1, ura3::HIS3::[pAFS152:URA3 P_CYC_- lacI-GFP], sec3::[pBK13.1:LEU2 lacO]* |
| TMS201 | *MAT***a***, his3-11,15, lys2-801::[pLL1.1: LYS2 Pr_CYC_GFP lacI], arg4-ΔHpa, , zip1::KANMX*  *S. carlbergensis* chromosome *V: URA3, pac2::[pD174:LEU2 lacO], ilv1* |
| TMS265 | *MAT***a***, his3-11,15, lys2-801::[pLL1.1: LYS2 Pr_CYC_GFP lacI], mad2::NAT, S. cerevisiae* chromosome *V: ura3::HIS3, ilv1, rad3, sec3::[pBK13.1:LEU lacO]* |
| TMS266 | *MAT****α****, ade1::ARG4 , his3-11,15, arg4-ΔHpa, mad2::NAT*  *S. cerevisiae* chromosome *V: rad3, ilv1, ura3::HIS3::[pAFS152:URA3 P_CYC_-GFP-lacI], sec3::[pBK13.1-LEU2-lacO]* |
| TMS275 | *MAT****α****, ade1::ARG4 , his3-11,15, arg4-ΔHpa, mad2::NAT*  *S. cerevisiae* chromosome *V: rad3, ilv1, ura3::HIS3::[pAFS152:URA3 P_CYC_-GFP-lacI], sec3::[pBK13.1-LEU2-lacO], zip1::KANMX* |
| DDO50-34c | *MATα, ura3-13, met13-d, ura3::[pAFS152:URA3 P_CYC1_-GFP-lacI], trp1-Δ63, leu2, tyr1-1, lys2-1, met13-d, can1, zip1::KANMX, CEN1::pJN2[lacO LEU2], MTW1-13xMYC-TRP1* |
| DDO51-35b | *MAT****a****, leu2, lys2-2, tyr1-2, met13-c, ura3::[pAFS152:URA3 P_CYC1_-GFP-lacI], trp1-Δ63, cyh2-1, his3-Δ1, CEN1:: [pJN2:lacO LEU2], MTW1-13xMYC-HIS3, zip1::KANMX* |
| DDO54.5-7b | *MATα, ura3-13, ura3::[pAFS152:URA3 P_CYC1_-GFP-lacI], trp1-Δ63, leu2, tyr1-1, lys2-1, met13-d, can1-R, spo11::KANMX, zip1::KANMX, CEN1::[pJN2:lacO LEU2], MTW1-13xMYC-TRP1* |
| DDO69-6d | *MAT***a***, ura3-13, trp1-Δ63, leu2, tyr1-1, lys2-1, met13-d, can1-R, his3-Δ1, P_CLB2_-3HA-CDC20 KANMX6, MTW1-13xMYC-TRP1, SPC42-[MDE1145: URA3 SPC42-DSRed]* |
| DDO70-1a | *MAT∝, leu2, lys2-2, met13-c, tyr1-2, ura3-1, trp1-Δ63, cyh2-1, P_CLB2_-3HA-CDC20 KANMX6, his3-Δ1, MTW1-13xMYC-HIS3, SPC42-[MDE1145: URA3 SPC42-DSRed]* |
| DDO74-22a | *MAT***a***, URA3::pKB81 [P_CYC1_-GAL4(848)-ER], trp1-63, his3-1, HIS7, leu2, met13-d, tyr1-1, lys2-1, can1 (R), + (S), KanMX-P_GAL1_-NDT80, MTW1-13xMYC-TRP1, SPC42-[MDE1145: URA3 SPC42-DSRed]* |
| DDO75-36c | *MAT∝, leu2, lys2-2, met13-c, tyr1-2, URA3::pKB81 [URA3, P_CYC1_-GAL4(848)-ER], trp1-Δ63, cyh2, HIS7, his3-Δ1, KanMX-Pgal-NDT80, MTW1-13xMYC-HIS3, SPC42-[MDE1145: URA3 SPC42-DSRed], pKB81 [URA3, P_CYC1_-GAL4(848)-ER]* |
| ABY194-Nd | *MATα, ura3-13, trp1-63, his3-1, HIS7, leu2, met13-d, ADE2, ADE5, lys2-1, ade1::LYS2, ura3::[pAFS152:URA3 P_CYC1_-GFP-lacI], CEN1::[pJN2:lacO LEU2], MTW1-13XMyc::TRP1 ade1::LYS2* |
| TSP58.1 | *MAT***a***, his3-1, 15, lys2-801::[pLL1.1: LYS2 Pr_CYCl_ lacI-GFP], arg4-ΔHpa, S. carlbergensis* chromosome *V: URA3, pac2::[pD174:LEU2 lacO], ilv1, zip2::KANMX* |
| THC14.1 | *MAT****α****, ade1::ARG4, trp2, leu2, his3-11,15, arg4-ΔHpa, lys1,*  *S. cerevisiae* chromosome *V: rad3, ilv1, ura3::HIS3::[pAFS152:URA3 Pr_CYC_- lacI-GFP], sec3::[pBK13.1:LEU2 lacO], zip2::KANMX* |
| THC19 | *MAT****α****, ade1::ARG4, trp2, leu2, his3-11,15, arg4-ΔHpa, lys1,*  *S. cerevisiae* chromosome *V: rad3, ilv1, ura3::HIS3::[pAFS152:URA3 Pr_CYC_- lacI-GFP], sec3::[pBK13.1:LEU2 lacO], zip3::KANMX* |
| THC22 | *MAT***a***, his3-1, 15, lys2-801::[pLL1.1: LYS2 Pr_CYCl_ lacI-GFP], arg4-ΔHpa, S. carlbergensis* chromosome *V: URA3, pac2::[pD174:LEU2 lacO], ilv1, zip3::KANMX* |
| TD623.1 | *MAT***a***, his3-11,15, lys2-801::[pLL1.1: LYS2 Pr_CYC_GFP lacI], mad2::NAT, S. cerevisiae* chromosome *V: ura3::HIS3, ilv1, rad3, sec3::[pBK13.1:LEU lacO], zip1::KANMX* |
| TD683.3 | *MAT∝, ura3::pAFS152[URA3 PCYC-GFP-lacI], his3-11,15, arg4-HpaI, lys2-801::[pLL1: LYS2 Pcyc1-lacI-GFP], sec3::[pBK13.1:LEU2 lacO], zip4::pFA6a KanMX* |
| TD685 | *MAT***a***, URA3, his3-11,15, lys2-801::pLL1[PCYC1-GFP-lacI LYS2], pac2::[pD174:LEU2 lacO], zip4::pFA6a KANMX6* |
| TLL1.16 | *MATα, ade1::ARG4, leu2-3,112, ura3::HIS3, trp2, his3-11,15, arg4-ΔHpaI, rad3, cyh2-1, ilv1-92, cup1::ura3::THR1, ura3::*[pAFS152: *URA3* Pr_CYC_-GFP-lacI] |
| DD654 | TMS275.4 x TD623.1 |
| DD728 | DMS46.7.3D x DMS143.16A |
| DD732 | THC14.1 x TSP58.1 |
| DD737 | THC19 x THC22 |
| DD770 | TLL1.16 x DMS351.4A |
| DDO45 | DDO54.5-7b x ABY194-Nd |
| DDO55 | DDO50-34c x DDO51-35b |
| DDO72 | DDO69-6d x DDO70-1a |
| DDO76 | DDO74-22a x DDO75-36c |
| DHC42 | TMS46-7.3D x TMS143.16A |
| DHC49 | TD683.3 x TD685.14 |
| DHC54 | TMS201 x DMS301.12D |
| DMS179 | DMS175.20A x DMS127-1.6A |
| DMS296 | TMS189-2 x MDY2196 |
| DMS321 | TMS187 x TMS196 |
| DMS371 | DMS350.11D x TMS46-7.3D |
| DMS372 | TMS46-7.3D x DMS143.16A |
| DMS381 | DMS320.14B x TMS199 |
| DMS382 | TMS199 x TMS201 |
| DMS383 | DMS348.42A x DMS377.18A |
| DMS384 | DMS377.29B x DMS347.2A |
| DMS387 | TMS265 x TMS266 |
|  |  |
