# Supplementary material for: The Synaptonemal Complex Protein Zip1 Promotes Bi-Orientation of Centromeres at Meiosis I
Source: PLoS Genet. 2009 Dec 11;5(12):e1000771. doi: 10.1371/journal.pgen.1000771 (PMC2781170; doi:10.1371/journal.pgen.1000771)
Supplement: Table S2 — Primers used for Chromatin Immuno-precipitation. (0.04 MB DOC) [file pgen.1000771.s002.doc]

**Table S2. Primers used for Chromatin Immuno-precipitation**

| Locus | Primer name | Sequence |
| --- | --- | --- |
| CEN4 | Cen4_449635F | ACATATATTACACGAGCCAG |
|  | Cen4_449943R | CTCGAACTGATCTATAATGC |
| CEN4-5kb | Chr4_444738F | CCATTAGCACAAATATGGCT |
|  | Chr4_445045R | CTTGTTACAGTGTTCAGTCT |
| CEN4-10kb | Chr4_ 439660F | AGCTCTCAAAGTTGTCTCAA |
|  | Chr4_439956R | ATGTCATATGGTGTTTCTGG |
| CEN4+5kb | Chr4_454779F | CATGACTTGC GGTATAGAAA |
|  | Chr4_455082R | GAGGATAATGTGTGAGAAGA |
| CEN4+10kb | Chr4_459815F | CAATTCTGTG TCCAGTACAT |
|  | Chr4_460128R | TGCAGGGCTTAAAAGAGATT |
| CEN5 | CEN5-151604F | CCTCCTAGCACTTCGTAATGTTTCTGTC |
|  | CEN5-151876R | TATTTTTGCTTGGCCCTTTGTTTACC |
